# Compression of X-ray Free Electron Laser Pulses to Attosecond Duration

**DOI:** 10.1038/srep16755

**Published:** 2015-11-16

**Authors:** James D. Sadler, Ricky Nathvani, Piotr Oleśkiewicz, Luke A. Ceurvorst, Naren Ratan, Muhammad F. Kasim, Raoul M. G. M. Trines, Robert Bingham, Peter A. Norreys

**Affiliations:** 1 Clarendon Laboratory, University of Oxford, Parks Road, Oxford OX1 3PU; 2 Central Laser Facility, STFC Rutherford Appleton Laboratory, Didcot, Oxon, OX11 0QX; 3 Department of Physics, University of Strathclyde, John Anderson Building, 107 Rottenrow, Glasgow, G4 0NG.

## Abstract

State of the art X-ray Free Electron Laser facilities currently provide the brightest X-ray pulses available, typically with mJ energy and several hundred femtosecond duration. Here we present one- and two-dimensional Particle-in-Cell simulations, utilising the process of stimulated Raman amplification, showing that these pulses are compressed to a temporally coherent, sub-femtosecond pulse at 8% efficiency. Pulses of this type may pave the way for routine time resolution of electrons in nm size potentials. Furthermore, evidence is presented that significant Landau damping and wave-breaking may be beneficial in distorting the rear of the interaction and further reducing the final pulse duration.

In recent years, outputs from facility scale X-ray Free Electron Lasers (XFELs) have set new boundaries for the brightest X-ray sources available, improving on synchrotron sources by at least nine orders of magnitude. Current facilities such as the Stanford Linac Coherent Light Source (LCLS)^1^ are greatly over subscribed and many new facilities are in development, such as the European XFEL^2^ in the DESY Laboratory in Germany and the the operational SACLA facility in Japan. The pulses generated by these machines have contributed to research areas as diverse as protein crystallography^3^, phase transitions^4^ and superconductors^5^.

The XFEL wavelength is variable from soft X-rays down to the Ångström level. Recent advances in seeding the emission process (with a low bandwidth precursor) and use of a low bunch charge will lead to a reduction in pulse duration to a few femtoseconds^6^,^7^. The peak brightness has been significantly improved by seeding^8^. Attosecond pulses can also be generated via interference with high harmonics^9^. Both of these techniques provide pulses of energy 10 *μ*J or less and previous experiments report the need for sub-femtosecond pulses with greater fluence^10^.

Many areas of science and technology measure and attempt to control electrons in 10 eV, nm scale potentials. Temporal evolution of chemical bonds, bio-information and silicon microchip transistors all fall in to this category. Temporal resolution is needed at the level 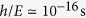
11. Energetic attosecond pulses are also desirable for better resolution in destructive scattering experiments where the timescale of molecular destruction in an intense X-ray beam is of the order 1 fs^12^.

This paper reports the first comprehensive computational study of Raman compression for XFEL pulses. This is achieved via stimulated Raman backward scattering (RBS), a proposal explored in reference^13^. This laser plasma interaction, in an underdense plasma, transfers some of the energy of a typical XFEL pulse to a much shorter sub-femtosecond pulse, as illustrated in Fig. 1.

Extensive analytical^14^,^15^,^16^,^17^ and computational studies^18^,^19^,^20^ of Raman amplification in the optical regime have shown that a pump laser pulse may transfer its energy to a much shorter counter-propagating seed pulse, finishing at up to 1000 times the pump pulse power. The same RBS process leading to asymmetries in hohlraum plasmas^21^ is here used for amplification.

Multi-dimensional simulations at infra-red wavelengths have shown efficiencies from 10% to over 30%, leaving output pulses in the petawatt regime^19^. However, a similar set-up with a Vlasov-Maxwell code has shown the potentially destructive effects of Langmuir wave-breaking on the efficiency^22^, also exhibited in 2D simulations using the Particle-in-Cell code EPOCH^23^. Proof of concept experiments generated gigawatt infra-red (IR) pulses^24^ and a further multi-pass design^25^ has shown that as the seed grows to greater intensity than its pump, the energy transfer continues towards the seed pulse.

Raman backward scattering can be stimulated by the counter-propagation of a pump pulse at frequency *ω*_0_ and a seed pulse at frequency *ω*_0_ − *ω*_*p*_ (where *ω*_*p*_ is the electron plasma frequency) and around 1/1000th the duration. The region where they overlap is an underdense plasma with electron density *n*_*e*_ around 1% of the critical density *n*_*crit*_. This means both pulses propagate almost as if in vacuum. The beating of these pulses resonantly excites an electron plasma (Langmuir) wave at frequency *ω*_*p*_ and approximate wavenumber (2*ω*_0_ − *ω*_*p*_)/*c*. The pump pulse then couples with this density perturbation and the lower Stokes component amplifies the seed, leading to a parametric instability with growth rate 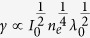, where *I*_0_ and *λ*_0_ are the pump intensity and wavelength respectively.

There exists a non-linear stage of the interaction^16^,^26^. The amplified pulse exhibits self-similar behaviour as its energy grows proportional to the interaction time and its duration decreases inversely proportional to its energy. This solution is an attractor, and describes the pulse evolution provided that electron-ion collisions, Langmuir wave-breaking and relativistic effects are negligible.

Furthermore, this solution is scale invariant provided that 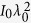 and *n*_*e*_/*n*_*crit*_ are kept fixed. Therefore as noted in reference^13^, their optimal results may be equally applicable to a 10 nm soft X-ray pump pulse. The required scaled pump intensity is 5 × 10^18^ W/cm^2^ and electron density 5 × 10^22^/cm^3^ (less than 1% of critical density). These are parameters directly accessible to an X-ray free electron laser focused to a spot size of 1–2 *μ*m, a feat achieved at shorter wavelengths by a group at the SACLA laser in Japan^27^. A typical pulse duration of 250 fs means the seed propagates through the pump pulse over 40 *μ*m and so this was chosen as the plasma thickness. This distance is over 30 times the characteristic RBS growth distance, so there is ample opportunity for the non-linear stage to be reached, even with a seed much less intense than the pump. The Rayleigh range exceeds the interaction distance by a factor of 10.

A seeded XFEL pulse will lead to a lower bandwidth and higher brightness^8^, these are favourable traits for an RBS pump as the process is sensitive to small deviations from frequency matching. Although brightness is often higher for shorter wavelength XFEL pulses, it is the dimensionless pump amplitude *a*_0_ that appears in the RBS growth rate and this will be higher for longer wavelengths.

One may use this scale invariance to assess the optimal pump pulse wavelength. Keeping *ω*_*p*_/*ω*_0_ fixed while scaling all distances linearly, the number of RBS e-foldings is 
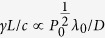
 where *P*_0_ is the peak power, *D* is the spot size and *L* is the interaction distance. Both the XFEL pulse power and the ratio *λ*/*D* will decrease as one goes to harder X-rays. Therefore high power long wavelength pump pulses of *λ* = 10 nm were considered.

However, one must be careful in using the scale invariance to extrapolate into this regime. Gas jet experiments at optical wavelengths generate a classical collisionless plasma at a density four orders of magnitude lower than the case considered here. At these higher densities, collisional damping of the pump and Langmuir waves will be much more pronounced. This can be seen by calculating the plasma parameter. This damping will lead to much higher temperatures in the plasma. By calculating the ratio of the Langmuir wave phase velocity to the electron thermal velocity, one sees that this also leads to prominent Landau damping:


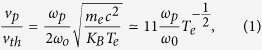



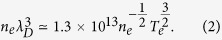


Here electron density is in units of cm^−3^ and electron temperature in units of keV. *λ*_*D*_ is the Debye length and all other symbols have their usual meanings.

The one-dimensional (1D) radiation hydrodynamic code Helios^28^ was initially used to estimate conditions in the plasma under irradiation by the focused pump pulse. A 40 *μ*m plastic (CH) plasma slab with density 5 × 10^22^ cm^−3^ and Spitzer resistivity was modelled with a 200 eV starting temperature. For a typical 100 fs irradiation at intensity 10^18^ W/cm^2^, almost uniform electron temperatures of 400 eV are predicted in the dense target plasma once the pump radiation had passed through. This leads to a value of 1.3 for (1) and a Maxwellian averaged Landau damping rate of 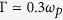. Collisions are also important on the timescale 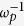 as the value of 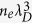 is 14–20.

One therefore concludes that the plasma wave will be heavily damped and in the so called “Quasi-transient regime”^29^ where the plasma wave damping exceeds the RBS growth rate. The damping reduces the effective RBS growth rate. Since the Landau damping and collisional damping require opposite conditions to minimise, we must accept a reduced RBS growth rate and expect lower efficiency than the studies at optical wavelengths.

As predicted by Malkin *et al.*^13^ there is a cut-off wavelength below which Raman amplification becomes unfeasible due to heavy collisional damping of the pump pulse and/or heavy Landau damping suppressing the plasma wave. From Malkin *et al.*’s analytical results, the cut-off is in the region of 1 nm.

All of these considerations indicate an optimal parameter window of *λ* > 1 nm, with a range of intensities and plasma densities shown in the results Table 1. This window was explored with 1D simulations performed using the Particle-in-Cell (PIC) code Osiris^30^. These plasma conditions are accessible to a PIC code including the various damping effects. Kinetic effects such as Landau damping and wave-breaking are included by default, whereas particle collisions are here modelled using Monte Carlo binary collision methods.

A fixed simulation window of width 45 *μ*m contained a uniform plasma of width 40 *μ*m. The pump wavelength was chosen as a compromise between better efficiencies at longer wavelengths but shorter final pulse durations at lower values. A constant intensity pump pulse of wavelength 10 nm and duration 250 fs counter-propagates with a transform limited Gaussian seed pulse of initial duration 1.5 fs full width at half maximum (FWHM), wavelength 11 nm and peak intensity equal to the pump intensity. Its wavelength was calculated via the frequency matching condition including the Bohm-Gross correction^31^:


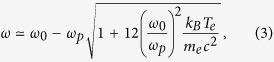


where a very underdense plasma is assumed. Both have aligned linear polarisation. For dense, non-classical plasmas the Langmuir wave frequency is further increased^32^. We expect these quantum corrections to be negligible in this regime and it was found in the simulations that equation (3) is sufficient for resonance and accurately describes the central frequency of the output.

Due to the collisional nature of the plasma, 1D simulations included a routine to model the electron-ion and electron-electron collisions, where relativistic effects are accounted for. The initial plasma temperature was 200 eV, with collisional damping causing a rise to around 500 eV after the pump has propagated through the plasma, in agreement with the 1D hydrodynamic simulations.

Table 1 shows the results of these 1D simulations. Currently the highest pump pulse intensities available are limited to the top row of Table 1, however one can see the performance dramatically increases for higher intensities due to the increased linear RBS growth rate.

Of course, other growing instabilities are also present. Lead among these are Raman forward scatter, producing longitudinal modulations to the pulse, and filamentation, producing a transverse breakup. These effects are a hindrance but there exists an optimal parameter window where the e-foldings of these unwanted instabilities are minimised while still giving effective amplification.

The focused intensities exceed the filamentation threshold set by Bingham *et al.*^33^ However their characteristic growth distance of the thermal filamentation for soft X-rays reduces to


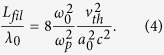


With *v*_*th*_ the electron thermal velocity and *a*_0_ the dimensionless vector potential, having the range 0.01–0.03 for these parameters. This suggests *L*_*fil*_ > 1000*λ*_0_, showing the interaction is over before heavy thermal filamentation sets in. In any case, thermal filamentation is of less concern for this application as the seed will not be subsequently focused as for equivalent schemes with IR pulses. The energy transfer efficiency and final pulse duration are the more important factors to optimise. In addition, Raman forward scatter is reduced to acceptable levels for *n*_*e*_/*n*_*crit*_ < 0.01.

To assess the importance of these competing instabilities, a successful 1D simulation was repeated in 2D, with the growth of seed intensity shown in Fig. 2. The smooth transverse profile agrees with the analytical estimate of low filamentation and the efficiency of 3.0% is consistent with the 3.8% found in 1D, showing that transverse effects have little impact even at this highest intensity.

Better focusing of the X-ray pulses, or future upgrades, will increase the growth rate and allow pulses to be compressed down to a few hundred attoseconds, retaining up to 8% of the pump energy. The performance is generally seen to increase with higher densities. However, it is best to keep to less than 0.6% of critical density to reduce the growth of competing plasma instabilities. Smoother pulse envelopes and lower prepulses (by a factor of 100) were observed for the simulations at lower plasma density.

The intensities here are slightly above the plasma wave breaking threshold^34^,^35^:


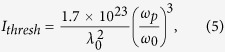


with the pump wavelength in units of nm and intensity in W/cm^2^.

Collisionless Vlasov-Maxwell simulations for Infra-red pulses have shown efficiency is markedly reduced by the effects of wave-breaking^22^ when the pump greatly exceeds this threshold. Here we intend an interaction in the mild wave-breaking regime. The distortion to the rear of the Langmuir wave will reduce the pulse duration even for low RBS growth, while the efficiency is only slightly reduced in the mildly over-threshold case^22^.

A further simulation with similar parameters but an initial seed of 10 times the pump intensity was used to verify this. Its electron phase space diagram, shown in Fig. 3, is plotted near the start of the interaction. The short wavelength Langmuir wave is excited right from the front of the seed pulse but only manages around 20 periods of oscillation before it breaks. This places an upper limit on the final duration of the seed of around 10 cycles. In addition, the distribution is skewed towards negative momentum, suggesting heavy Landau damping from the plasma wave travelling at −0.03*c*. From the position of the seed pulse FWHM, one can see that both of these effects will cease amplification near the back of the seed pulse and help to shorten it, as the front is amplified more than the rear.

Although wave-breaking reduces the efficiency, experiments require the shortest possible pulse with usable photon content. As this condition is met for these mJ pulses, it is best to shorten the duration at the cost of energy content and so wave-breaking may actually be considered beneficial^36^. Indeed, the evolution of the pumped pulses from Table 1 does not follow the self similar behaviour too well. The pulse remains shorter than predicted by this model at all times.

In further agreement with reference^29^, the secondary pulses of the output are suppressed to less than 1/100 of the primary pulse intensity, compared with around 1/20 in the case with negligible damping.

In a further simulation scaled for a wavelength of 1 nm at constant 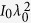, the efficiency was comparable to the 10 nm case. However a typical XFEL pulse has lower power at shorter wavelengths. A second simulation with realistic parameters showed lower coupling to the seed (by a factor of 10) but still produced coherent sub-femtosecond radiation. In general, the efficiency is found to be better for longer wavelengths. A direct comparison between the 10 nm and 1 nm cases reveals slight pump absorption and consequently higher temperatures of up to 1 keV in the second case.

It is expected that a high harmonic seed will initially be orders of magnitude less intense than its pump pulse. As also predicted in reference^29^, this will reduce the efficiencies below those of Table 1. This was verified in a repeat of the highest efficiency case from Table 1, with a more realistic seed intensity of 10^16^ W/cm^2^. However, the wave-breaking threshold is independent of the initial seed intensity and so this effect still shortens the seed to 500 as, albeit at the lower efficiency of 1.5%.

The best results were found for plasmas below solid density and so an initial optical pre-pulse may be used to expand and heat a plastic target to the conditions simulated, thus minimising the X-ray opacity while keeping the RBS growth rate high.

The results of this study indicate a feasible route to attosecond pulses with XFEL like energies. Experiments with relativistic plasma irradiation by infrared pulses have already yielded high harmonics into the X-ray band^37^,^38^,^39^,^40^,^41^, giving a promising candidate for a seed pulse. The spatial extent of these harmonics will ease requirements for precision alignment. In addition, the high repetition frequency of harmonic radiation eases the required temporal synchronisation with the XFEL pulse. Because the seed radiation contains a large number of harmonics, there will always be some photons within the bandwidth of the RBS amplification and so precise consideration of the seed wavelength is not needed. One could also imagine a second XFEL source in low charge mode to provide a 2 fs seed already at high power.

The wave interaction causes only minor depletion of the pump pulse and so a train of harmonic pulses may propagate through the pump one after the other and be amplified to nearly the same level. High repetition rate pulses of 300 as duration, with photon content typical of fourth generation sources, would open new frontiers in the study of ultra-fast processes.

## Additional Information

**How to cite this article**: Sadler, J. D. *et al.* Compression of X-ray Free Electron Laser Pulses to Attosecond Duration. *Sci. Rep.* **5**, 16755; doi: 10.1038/srep16755 (2015).

## Figures and Tables

**Figure 1:**
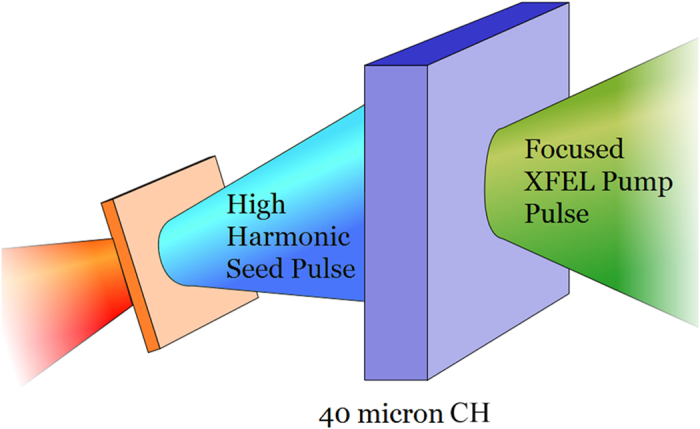
A schematic of the simulated set-up. A pump pulse of wavelength greater than 1 nm is focussed to highest possible peak intensity (>10^18^ W/cm^2^) on a target around 1/10 of solid density. A 2 fs seed pulse at slightly longer wavelength counter-propagates with a sufficiently small angular offset, such that the pulses interact for tens of microns. Under the conditions described, material absorption is low, whereas the plasma wave interaction depletes around 10% of the pump energy, with a portion of this scattered into the seed pulse as shown in Table 1. The interaction further reduces the seed duration to 500 as or less. The optimal pump pulse length is twice the width of the target, with linear polarisation for both pulses.

**Figure 2:**
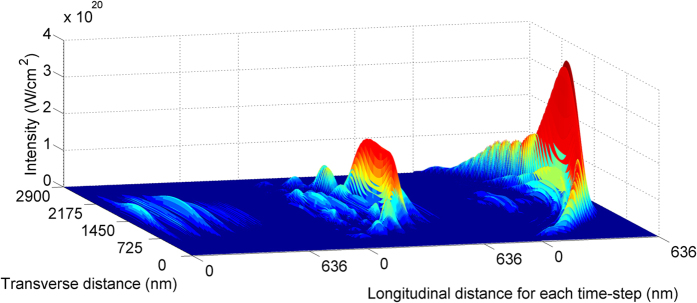
Three time-steps from a two-dimensional (2D) Particle-in-Cell simulation. The X-ray seed intensity increases as it propagates to the right, through the pump pulse (almost invisible on this scale) in the opposite direction. Time steps are after 16%, 70% and 100% of the 40 *μ*m interaction distance, containing a plasma with electron density 5.7 × 10^22^/cm^3^. The pump is 250 fs long, 10 nm wavelength and has constant intensity 1.2 × 10^19^ W/cm^2^. The initial seed is Gaussian transform limited with duration 1.5 fs and intensity equal to that of the pump. The emerging radiation has been compressed to 300 as and received 3% of the pump energy. There were 120 cells per pump wavelength, each with a width of 2 nm, initialised with 50 electrons and 5 ions per cell. Boundary conditions were free space. Distance between successive time steps is not to scale.

**Figure 3:**
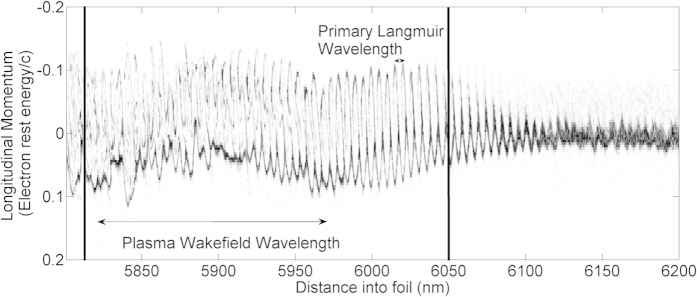
Electron phase space diagram for a 1D PIC simulation after 15% of the interaction distance. The FWHM of the seed pulse is enclosed by the solid black lines. The initial Maxwellian distribution is excited to a Langmuir wave which subsequently breaks within the extent of the seed pulse.

**Table 1:** Results for 1D PIC simulations with a pump wavelength of 10 nm and fixed duration 250 fs.

Pump Intensity (×10^18^ W/cm^2^)		Electron Density (×10^22^/cm^3^)			
		3.4	5.7	7.7	11
1.4	Duration (as)		1400	1280	
	Energy (mJ)		0.04	0.06	
	Efficiency		1.1%	1.7%	
5.5	Duration (as)		370	400	480
	Energy (mJ)		1.1	0.4	1.0
	Efficiency		8.0%	3.5%	7.0%
12.3	Duration (as)	330	290	260	
	Energy (mJ)	0.7	1.2	1.1	
	Efficiency	2.4%	3.8%	3.7%	

For each simulation, the following output pulse parameters are given: Full width at half maximum duration (as), energy content assuming 1 *μ*m spot size (mJ) and energy transfer efficiency from the pump pulse.
